# Serial evolutionary networks of within-patient HIV-1 sequences reveal patterns of evolution of X4 strains

**DOI:** 10.1186/1752-0509-3-62

**Published:** 2009-06-16

**Authors:** Patricia Buendia, Giri Narasimhan

**Affiliations:** 1 Department of Biology and Center for Computational Science, University of Miami, Coral Gables, FL, 33146, USA; 2 Bioinformatics Research Group (BioRG), School of Computing and Information Sciences, Florida International University, Miami, FL, 33199, USA

## Abstract

**Background:**

The HIV virus is known for its ability to exploit numerous genetic and evolutionary mechanisms to ensure its proliferation, among them, high replication, mutation and recombination rates. Sliding MinPD, a recently introduced computational method [1], was used to investigate the patterns of evolution of serially-sampled HIV-1 sequence data from eight patients with a special focus on the emergence of X4 strains. Unlike other phylogenetic methods, Sliding MinPD combines distance-based inference with a nonparametric bootstrap procedure and automated recombination detection to reconstruct the evolutionary history of longitudinal sequence data. We present serial evolutionary networks as a longitudinal representation of the mutational pathways of a viral population in a within-host environment. The longitudinal representation of the evolutionary networks was complemented with charts of clinical markers to facilitate correlation analysis between pertinent clinical information and the evolutionary relationships.

**Results:**

Analysis based on the predicted networks suggests the following:: significantly stronger recombination signals (p = 0.003) for the inferred ancestors of the X4 strains, recombination events between different lineages and recombination events between putative reservoir virus and those from a later population, an early star-like topology observed for four of the patients who died of AIDS. A significantly higher number of recombinants were predicted at sampling points that corresponded to peaks in the viral load levels (p = 0.0042).

**Conclusion:**

Our results indicate that serial evolutionary networks of HIV sequences enable systematic statistical analysis of the implicit relations embedded in the topology of the structure and can greatly facilitate identification of patterns of evolution that can lead to specific hypotheses and new insights. The conclusions of applying our method to empirical HIV data support the conventional wisdom of the new generation HIV treatments, that in order to keep the virus in check, viral loads need to be suppressed to almost undetectable levels.

## Background

One of the earliest and most striking observations made about HIV was the extensive genetic variation that the viral population exhibits within individual hosts, particularly in the hypervariable regions of the *env *(envelope) gene that code for the gp120 protein. Some of the remarkable properties of within-patient HIV quasispecies are their large population size, high replication rate, and short generation time. HIV has a high mutation rate (0.2 errors per genome per cycle) and an even higher recombination rate (3 events per genome per cycle) [2]. In North America, Western Europe, and Australia, the spread of HIV/AIDS has slowed down due to the introduction of multiple prevention and treatment strategies; however, in developing countries there are often not enough resources to combat the epidemic [3]. During HIV-1 infection a severe depletion in CD4+ T-Cells is characteristic of the onset of AIDS. Several studies have also shown a direct correlation between CD4+ decline and the proliferation of X4 strains, which lead to multinucleated cells called syncytia and to cell death [4]. Recent studies have shown a switch to X4 strains in viral reservoirs during effective highly active antiretroviral therapy [5]. The current study investigated a possible link between recombination and the emergence of the X4 strains.

Conventional phylogenetic programs are constrained to produce simple branching trees that can lead to misinterpretations of the phylogenetic relationships if the data set contains recombinants. One main assumption of most phylogenetic methods is that there is only one phylogeny underlying the evolution of the taxa under study. Recombination violates this assumption by generating mosaic genes, where different regions have different phylogenetic histories [6-8]. Furthermore, these traditional phylogenetic methods assume that all the sequence data are from contemporaneous taxa, which is not valid for serially-sampled data from longitudinal studies. Several methods that estimate the phylogenetic relationship of serially-sampled data have been published since 2000 [1,9-14]. However, only one of these methods takes recombination into account: Sliding MinPD [1]. The recombination process in HIV uses the mechanism of copy-choice replication, in which the viral polymerase switches between different RNA templates during transcription to give rise to mosaic genomes [15]. Mosaic genomes and genes can be identified through a computational sliding window method like the one implemented in Sliding MinPD. Sliding MinPD, the computational tool used in the current study, offers a choice of three different recombination detection methods (based on pairwise distances or topology comparisons) and calculates the statistical significance of the predictions (in terms of nonparametric bootstrap values) to infer the evolutionary history of a set of serially-sampled DNA or RNA sequences [1,16]. A recent study concluded that the variability found in a within-patient HIV population is due to a high incidence of recombination events [17]. Other studies have looked at the pervasive evolutionary force of HIV-1 recombination *in vivo *[18] and its effect on the emergence of drug-resistance [19,20]. A better understanding of the dynamics of HIV recombination is vital to the development of more complete models of HIV evolution that explain the escape of the virus from adaptive immunity and antiviral therapies.

In 1999, a comprehensive study of *in vivo *HIV evolution was published [21]. The study performed phylogenetic and statistical analysis on viral sequence data from the *env *gene collected at recurring time intervals from nine patients over a span of 8 to 12 years. The study provided new insights [21], but did not look at the effects that recombination has on the *in vivo *evolution of HIV. Henceforth we will refer to the HIV data set as the Shankarappa99 data set. We used Sliding MinPD to analyze the Shankarappa99 data set using data for eight of the nine patients for which hand-curated alignments were made available.

In this paper, we explore the discovery power of our computational tool by taking a closer look at the generated serial evolutionary networks. We present eight evolutionary networks obtained by applying Sliding MinPD to the Shankarappa99 data set and describe how the network information facilitates the study of viral evolutionary relationships, evolutionary patterns, splitting and merging of lineages, and how it helps to determine how these correlate with the disease status of the patient. Our analysis provides insight into within-host viral evolution and helps to find patterns that may explain the emergence of harmful mutants associated with disease progression.

## Methods

### Serial Evolutionary Networks

In a 1992 study, Holmes et al. created an "evolutionary framework" to express the inferred ancestor-descendent relationships in HIV sequence data that was serially sampled from a single patient [22]. The authors stated that "unlike most molecular phylogenies, real ancestors may be present in the data and the framework expresses the postulated ancestor-descendent relationships". Sliding MinPD, which is based on the above assumption, was used to construct "evolutionary networks" of 8 patients from the Shankarappa99 data set. Sliding MinPD combines a minimum pairwise distance (MinPD) approach, a sliding window method, and automated recombination detection to study the ancestor-descendant relationships of serially-sampled nucleotide sequences. In Sliding MinPD the identification of recombinants, ancestors and breakpoints is automated with no need for user intervention. It uses non-parametric bootstrap to measure the statistical support of its predictions. Three recombination detection options are available in Sliding MinPD (RIP, SB, and B-RIP) and have been previously described [1,16]. Briefly, Sliding MinPD takes as input a set of time-dated aligned sequences. The nucleotide columns in the alignment are bootstrapped into x replicates using a method in which some columns are included one or more times and others are omitted. The corrected distance matrix is calculated for the x replicates. The process is repeated using a sliding window approach that captures overlapping intervals of contiguous columns in the alignment. A recombinant sequence is identified when for different windows in the alignment different sequences are identified as ancestors with high bootstrap support. The method for recombinant identification varies slightly between RIP, SB and B-RIP. For the Standard Bootscan method (SB), for example, phylogenetic NJ trees are inferred and the topological distance between ancestor and descendant sequences is computed. Finally, NJ trees are constructed for all sets of sequences that share the same ancestors. Sequences identified as recombinants have two or more predicted ancestors and are included in two or more different NJ trees. More information can be found at the program's web site: 

### Data sets

**Figure 1:**
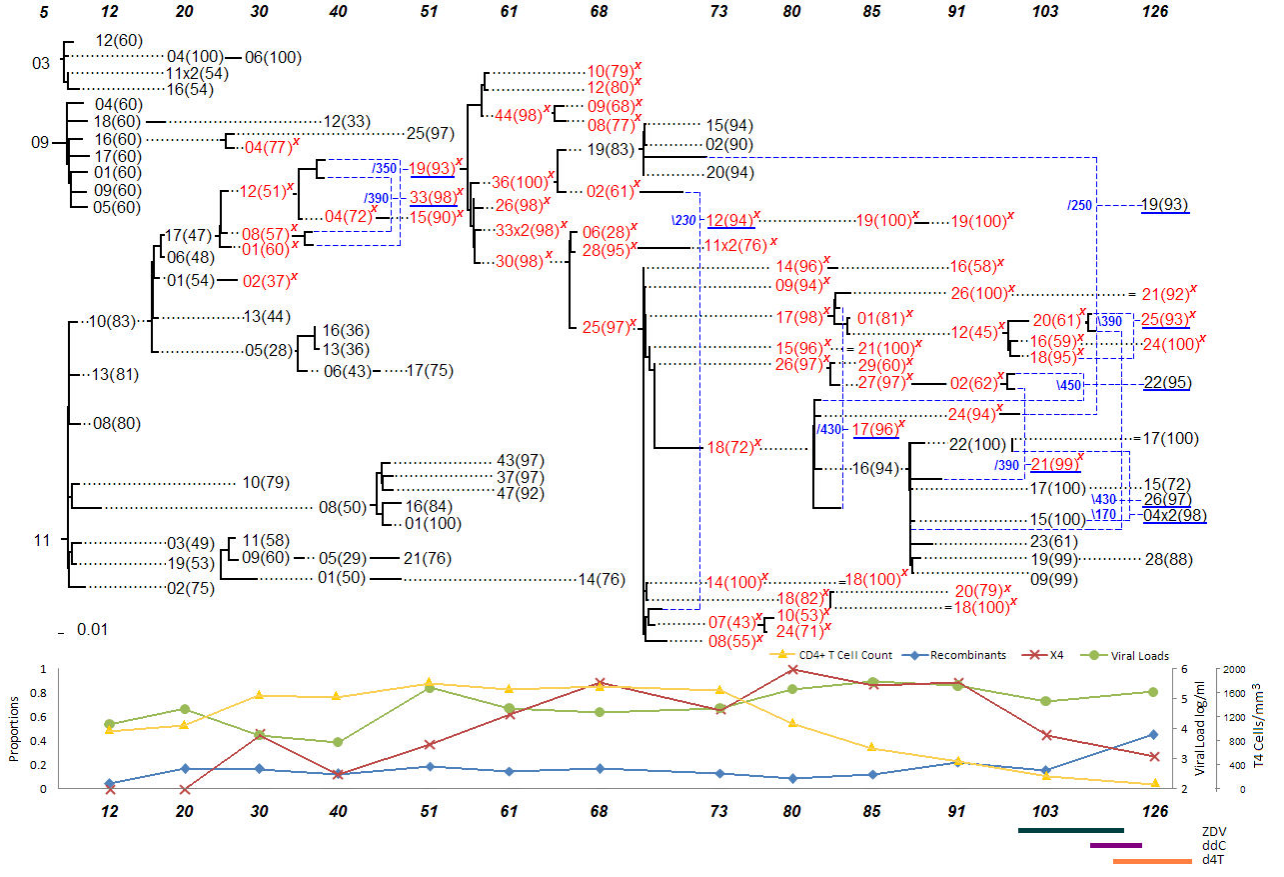
**Patient 2**.

The Shankarappa99 data set is available for download from GenBank and the HIV Los Alamos database. It consists of viral DNA and plasma RNA sequences from the C2-V5 region of the *env *gene sampled serially from patients with a moderate or slow rate of disease progression. All PCR amplifications were done with procedural safeguards to prevent recombination from occurring during PCR [21]. Two hand-aligned versions of the data set for 8 of the 9 patients were downloaded from the URL: , where it is available as supplementary material for a study on immune-mediated positive selection driven by HIV-1 molecular variation [23]. Both versions were available in the PAUP Nexus format and were separated into subsets, each corresponding to a single patient. One version was a gapped, hand-aligned file of the entire Shankarappa99 data set, where gaps signified insertions or deletions [23]. The length of the sequences was 786 nucleotides. The second data set contained a gap-balanced alignment of each of the subsets of the same data. Gaps had been removed from this data set in a "balanced" manner, i.e., such that codon alignments were preserved. Both the data sets, gapped and gap-balanced, had been aligned against reference HIV sequences (HIV-1 type B accession numbers K03455, M17451, U63632, and U21135) from the Los Alamos database. The sampled sequences correspond to amino acid positions 267 to 472 of the *env *gene of the reference sequences. The data sets of the 8 patients were then analyzed using Sliding MinPD. Duplicate sequences from the same sampling point were removed as they represented the same ancestral sequence, retaining just one copy, but marked with an x followed by the number of duplicate copies. Thus, the sequence labeled "11 × 2" from Figure 1 refers to two copies of sequence 11 from the sampling time point 20.

The sequences in the Shankarappa99 data set from the same time point had a relatively low level of diversity (average genetic distance: 0.03) and a low level of divergence relative to an initial founder sequence from the first sampling period (average genetic distance: 0.05). The exceptions were the data sets for patient 9 at all sampling points, and the sequences for patients 2, 3, and 7 for the last sampling times [21]. Low divergence causes a drop in performance in most recombination detection methods [24] and as described below we adjusted Sliding MinPD's settings to counteract those effects. Previous simulation studies and ROC curve analyses showed a recombination prediction performance of >0.9 for the bootstrap methods SB and B-RIP. Our simulation studies also showed a decrease in sensitivity for data with low divergence; however, the mean specificity remained above 0.97. The method proposed here is based on identifying the recombinant predictions which are supported by the three methods. We therefore trade in sensitivity for a high specificity value because a high confidence recombination prediction is desirable for visual analysis purposes and because sequences with some but not high enough recombination support will still be linked to one of the ancestral donor sequences.

We provided the alignment files, two for each patient, as input to Sliding MinPD, which ran each file using the three different recombination detection options: B-RIP (Bootscan RIP), SB (standard Bootscan) and RIP, producing 6 result files for each patient. In a final step, a consensus file was manually created by combining information from the 6 result files using a consensus strategy and a keen awareness of the strengths and weaknesses of the three methods used [1].

### Program Settings and Consensus Network

The settings for each option of the program were the same as the default options presented previously [1], with the exception of the RIP program, which was executed with a window size of 200 and a step size of 50, to compensate for the low divergence rates in the data sets. The default settings were as follows – window size = 200, step size = 20, bootstrap replicates = 100, bootstrap threshold = 90, PCC = 0.2 (for SB) and 0.4 (for RIP and B-RIP), corrected distance = TN93, rate heterogeneity alpha shape parameter = 0.5, and a bound of 1 for the number of crossovers. The results for non-recombinant predictions were less compromised given that MinPD (the predecessor of Sliding MinPD's RIP option) has been proven to perform well for non-recombinant data with low mutation rates [25].

A consensus file of ancestor descendant relationships was created manually from the 6 result files by applying the following guidelines in sequential order (if a rule was not applicable, the next applicable rule was chosen):

▪ Majority rule: The relationship chosen by a majority (at least four) of the six methods was chosen as the consensus.

▪ Consensus by bootstrap support: The result with the highest bootstrap value was chosen.

Consensus by priority: The results using the B-RIP option were given highest priority because of its superior performance and because it outputs the statistical bootstrap support for each estimate [1]. The results by SB had the second highest priority when choosing recombinant relationships. When all options agreed on a non-recombinant relationship, RIP was given the second highest priority for the ancestor choice. The results for the gap-balanced data were given secondary consideration for recombinant events, as the length of the alignment was often different from that of the gapped alignment and breakpoint positions could not be easily matched.

A serial evolutionary network was constructed for each of the 8 patients from the consensus file containing the ancestor-descendant relationships. A short summary of the guidelines used for interpreting the networks is given below:

▪ The sampling times in units of "months" from the time of seroconversion are shown at the top of each network. Thus, all sequences from the same sampling time are aligned vertically under the corresponding time.

▪ Full lines indicate branch lengths. Thus longer full lines indicate greater evolutionary distance. Dotted lines indicate linking relationships. Finally, dashed blue lines indicate recombination-linking relationships between donors and recombinants.

▪ Recombinant sequences are underlined in blue.

▪ Breakpoint positions appear after a slash at the point were the lines intersect. If the left donor is at the top (bottom, respectively) end of the recombination-linking dashed line, this is indicated by a forward (backward, respectively) slash followed by the breakpoint position. To locate a breakpoint position along the C2-C5 region of the *env *gene, it is necessary to know the coordinates of the gapped sequences with regards to the *env *gene map. The approximate start coordinates {within *env *gene| within HIV genome} of the *env *regions covered by the sequences are: pos 1 – C2:{799|7023}, pos 98 – V3:{891|7115}, pos 254 – C3:{995|7219}, pos 443 – V4: {1150|7374}, pos 579 – C4:{1243|7467}, pos 712 – V5:{1367|7591}, pos 772 – C5:{1403|7627}.

▪ X4-mutants appear in red and are also marked with a top-right elevated small x, as in the taxon 10(79)^x ^from Figure 1 at 68 months.

▪ An equal sign "=" next to a node indicates that the sequence is identical to an ancestral sequence from a previous sampling period. For example, the sequence 18(100) of patient 2 at 91 months is identical to sequence 18(82) from the 80 months sampling period.

▪ Statistical bootstrap support values, which provide statistical significance scores for the ancestor-descendant predictions are interpreted as follows:

◦ The bootstrap values are added in parenthesis after the sequence ID.

◦ The bootstrap values correspond to the ancestor-descendant relationship and not to the clades or topological position in the subtrees. The bootstrap value represents the support given to the choice of linking a specific query sequence to the ancestor at the root of the immediate subtree. Thus, the taxon labeled 43(97) at 61 months in Figure 1 corresponds to a bootstrap value of 97 for the ancestor-descendant relationship to taxon 08 at 40 months.

◦ Bootstrap values of recombinant sequences were obtained from computations involving only a few sequences from the pool of donor candidates.

◦ Unlike the bootstrap values of recombinant sequences, the bootstrap values of non-recombinant sequences were calculated by using all sequences up to the previous sampling point of the query sequence.

The sequence IDs have been shortened to decrease the amount of clutter and to make space for the bootstrap values. In their shortened form, they are unique within a sampling point, but not across the whole serial evolutionary network. For example, the GenBank ID of p2c005-03 is shortened as 03 in the network of patient 2 under the sampling time 5 months

## Results

Evolutionary networks for three of the eight patients who survived after the end of the analysis were discussed in detail. The statistical analysis section below discusses results based on all eight data sets and networks. In a previous study [14], the network of patient 2 (labeled as patient S in that study), was constructed using the MinPD method from that paper. In this paper, the serial evolutionary networks, which were produced by the improved method, Sliding MinPD, are presented. A mixture of the Genbank notation described above and the notation shown in the networks is used to refer to the patient's sequences: a sequence from patient 2 (Figure 1) defined by 12 m-10(83) appears as 10(83) under the 12 months column. The information about the health status of the patients, the therapy administered to each, viral loads and CD4+ T-cell counts were obtained from a previous study [21]. In addition to the serial evolutionary network, whose representation is described in detail in the previous section, each patient network is accompanied by a chart at the bottom displaying clinical markers (T-cell counts, viral loads), evolutionary markers (proportion of recombinants) and genetic markers (proportion of X4 sequences). Underneath the chart, the duration of drug therapy administration and the kinds of drugs that were prescribed are shown as horizontal bands under the time axis. The placement of this information under the serial evolutionary networks facilitates the visual discovery of patterns and associations between the different factors affecting the within-patient evolution of HIV.

### Patient 2

The Sliding MinPD analysis resulted in the following inferences and observations: Three different lineages were inferred up to the 30-months sampling time point. At that point, the lineage of 5 m-03 (with a 5 months start) became extinct. At 51 months the 5 m-09 lineage ceased to exist with sequence 51 m-25(97) as its last sampled sequence. Of more relevance is the splitting of the 5 m-11 lineage into several lineages, with strain 20 m-17(47) inferred as an early ancestor of the large X4 population that emerged after the 51 months period. All other arms of the 5 m-11 lineage disappeared within 68 months with the last sampled representative being 68 m-14(76). The evolutionary network made it convenient to understand how widespread the X4 genotype is in each sampling period. In particular, the most recent common ancestor of all post-61-months sequences carrying the X4 mutation was identified as 51 m-91(93), a recombinant sequence. The network suggests an increase in recombination activity in the last sampling period, which coincided with a decrease in the number of CD4^+ ^T-cells and the start of the antiretroviral drug therapy at the 103rd month period. The patient took three different drugs that included Zidovudine (ZDV), between 97 and 120 months, dideoxycytidine (ddC), between 109 and 125 months, and Stavudine (d4T), after 114 months. An interesting recombination event was suggested for sequence 126 m-19(93) with a putative left-side donor of 68 m-19(83), which was sampled almost 5 years earlier (a possible reservoir virus), and which was from a different lineage that did not carry the X4 mutation. This suggests the existence of dormant viruses that escaped latency and recombined with viruses from the dominant population. Putative dormant strains might enter the blood stream through the low degree of ongoing viral replication that is thought to occur in latently infected, resting CD4^+ ^T cells [26,27].

### Patient 8

**Figure 2:**
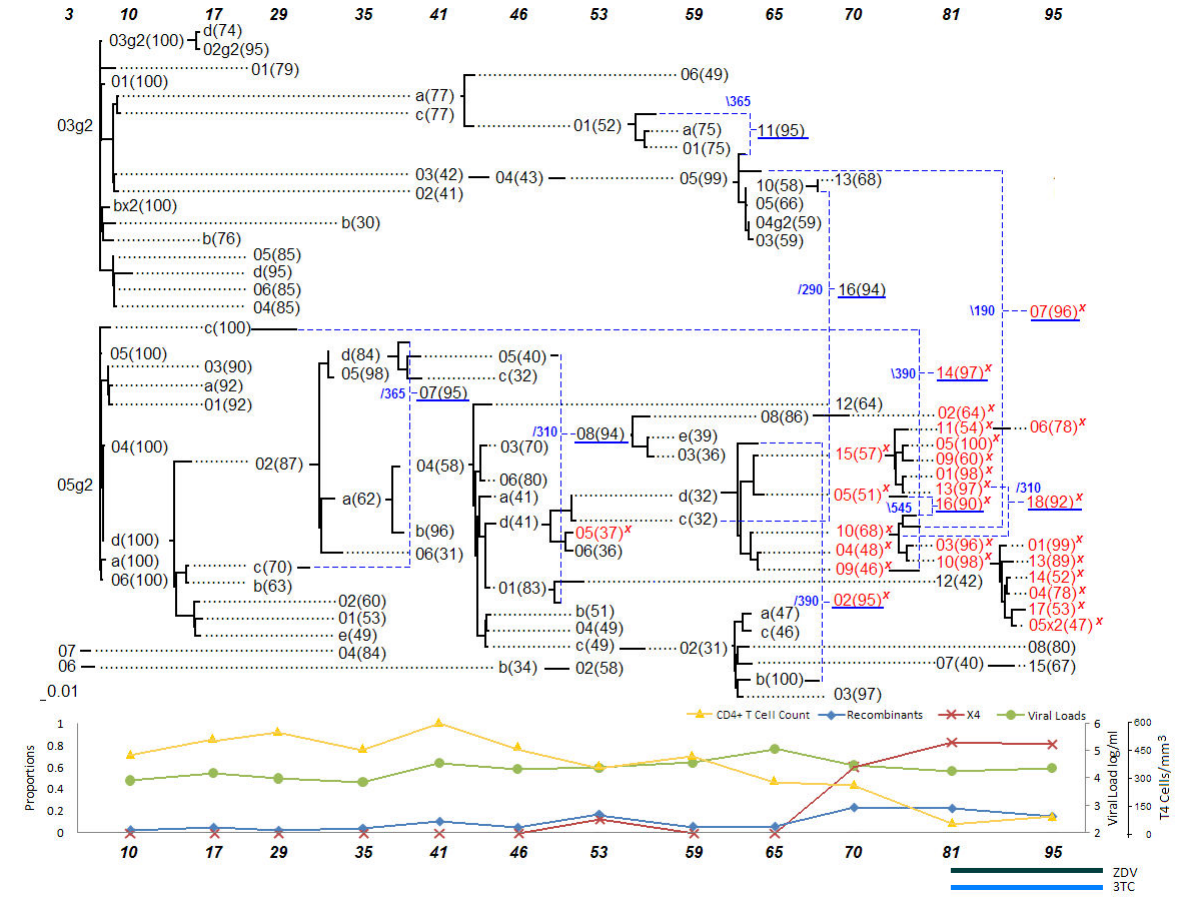
**Patient 8**.

The network of patient 8 as shown in Figure 2 also suggests the resurgence of reservoir viruses as suggested by the inferred recombinant sequence 81 m-14(97). The putative right-hand donor 17 m-c was sampled over 5 years earlier and does not have any sampled direct descendants that inherited the whole length of its sequence. The recombination event generating the sequence 95 m-07(96) joined two separate lineages, with the upper lineage ending at 70 months with sequence 70 m-13(68). Most recombinant sequences from 70 months and later had one donor that was from a different lineage. Patient 8 started on antiretroviral therapy at the 81 months period, the second to last sampling time, after a drastic decline in the number of CD4^+ ^T-cells, but no increased recombination effect could be observed.

### Patient 9

**Figure 3:**
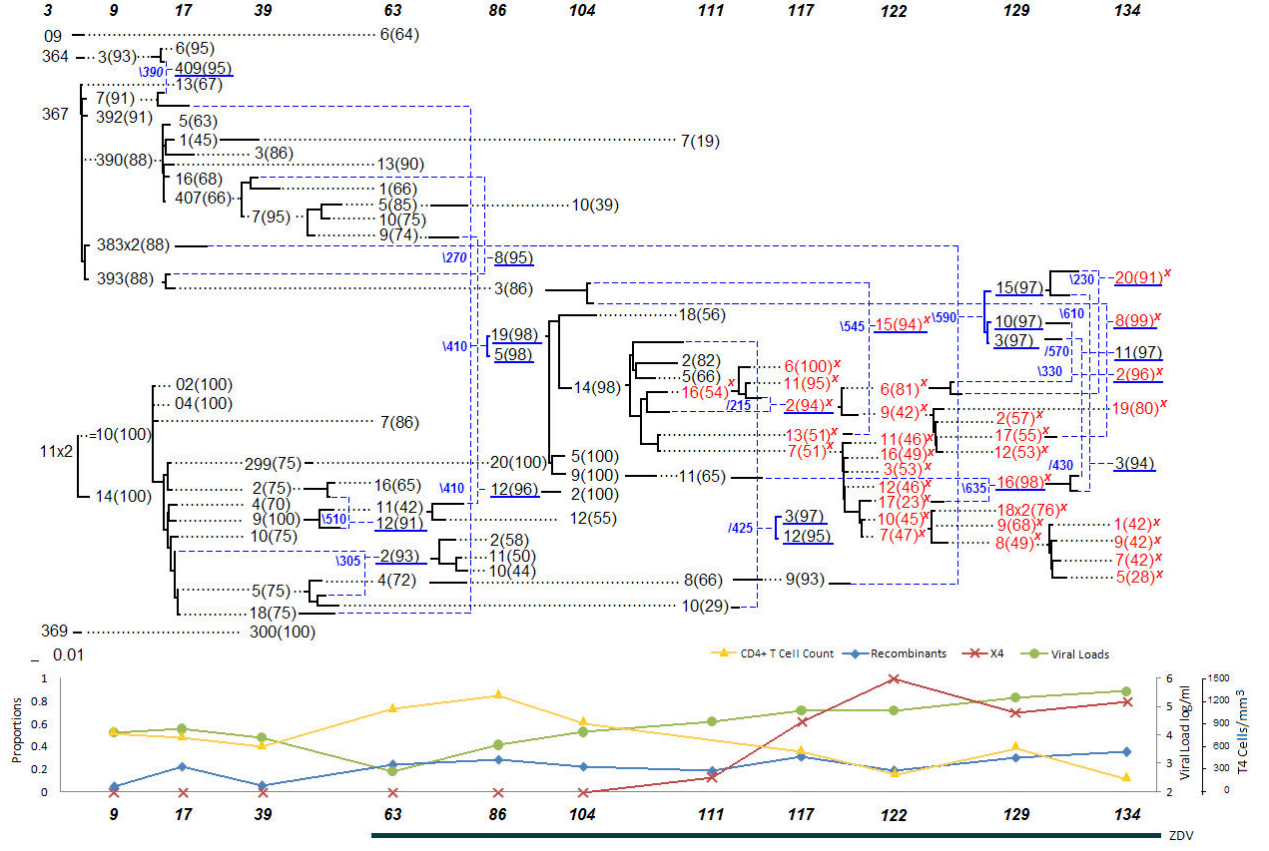
**Patient 9**.

As with patient 8 and 2, the network of patient 9 (shown in Figure 3) suggests the resurgence of reservoir virus as indicated by the recombination events at 129 months. Sequences 9 m-383 × 2(88) (with 2 identical copies) and 9 m-393(88) and its progeny 86 m-3(86) are predicted to be putative dormant strains that escaped latency. This pattern of dormant viral strains involved in recombination events with strains from later periods will be discussed in more detail in the following sections.

One aspect that has become apparent is the elevated number of recombinants among the X4-mutants, some of which produce strains without the X4 mutation. As previously observed with patient 2's network, the earliest putative ancestor of the X4 lineage is a recombinant itself, namely the sequence 86 m-19(98), and has predicted donors from two separate lineages, one that ceased to exist at 111 months and the other that persisted in recombination events through 134 months. Yet another feature of interest is that many recombinant sequences in patient 9 are themselves putative parental donors contributing to other recombination events. Patient 9 was on Zidovudine (ZDV) therapy since a doctor's visit at 57 months and was the only patient (among the ones considered in this work) with data spanning a long period of therapy of over 6 years.

### Statistical Analysis

**Figure 4:**
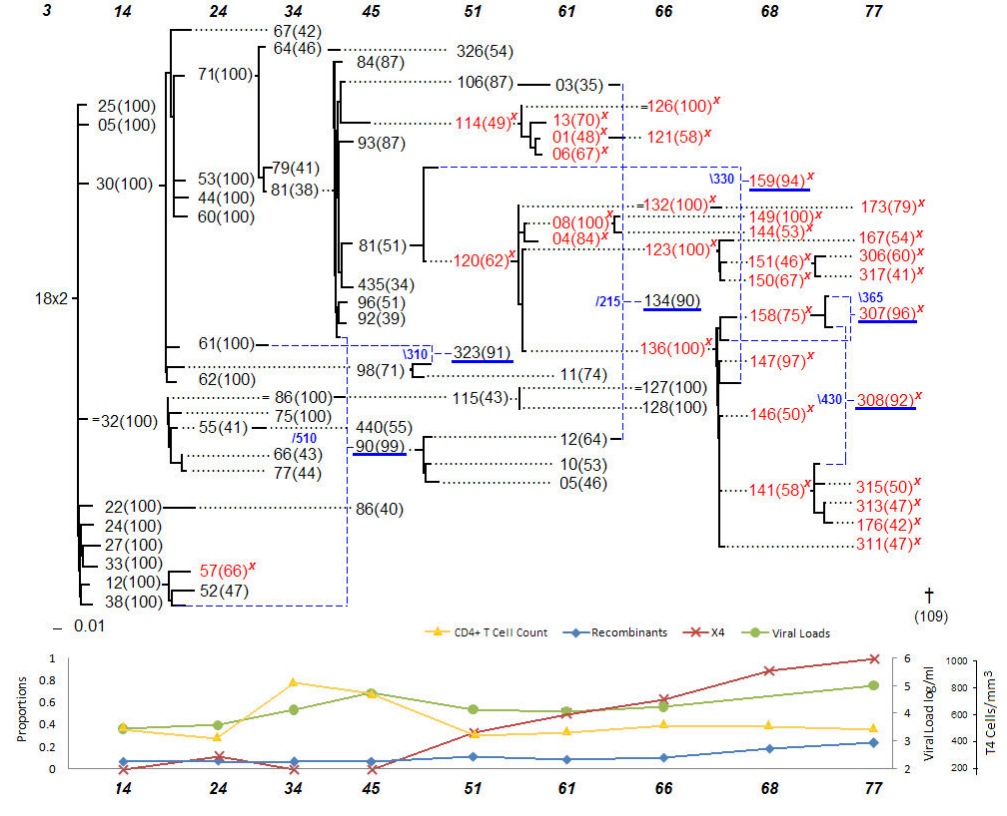
**Patient 1**.

**Figure 5:**
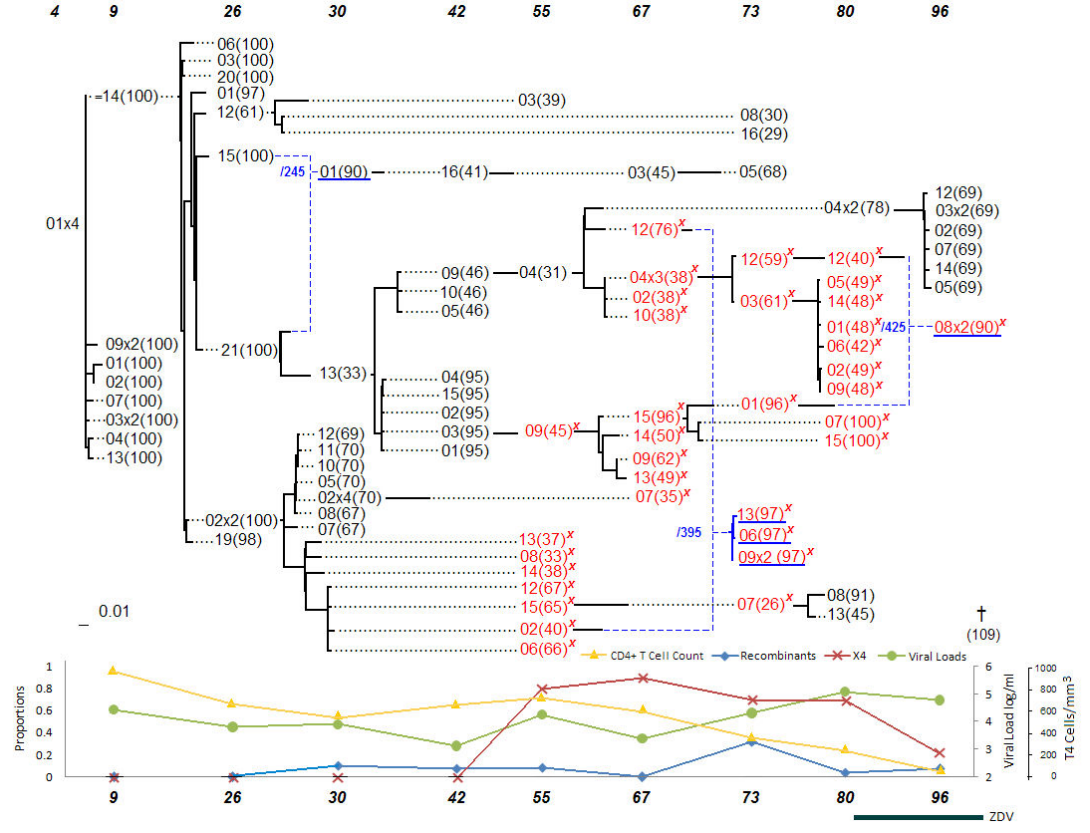
**Patient 3**.

**Figure 6:**
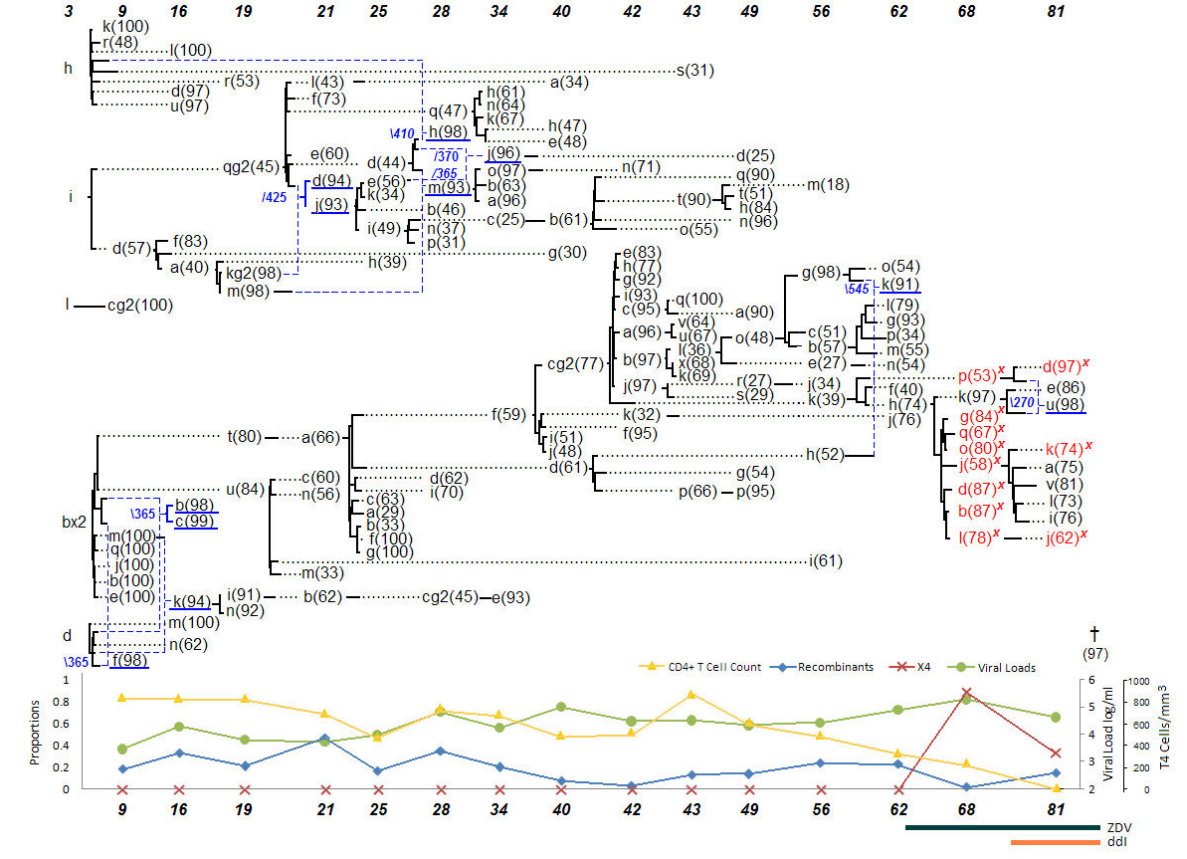
**Patient 5**.

**Figure 7:**
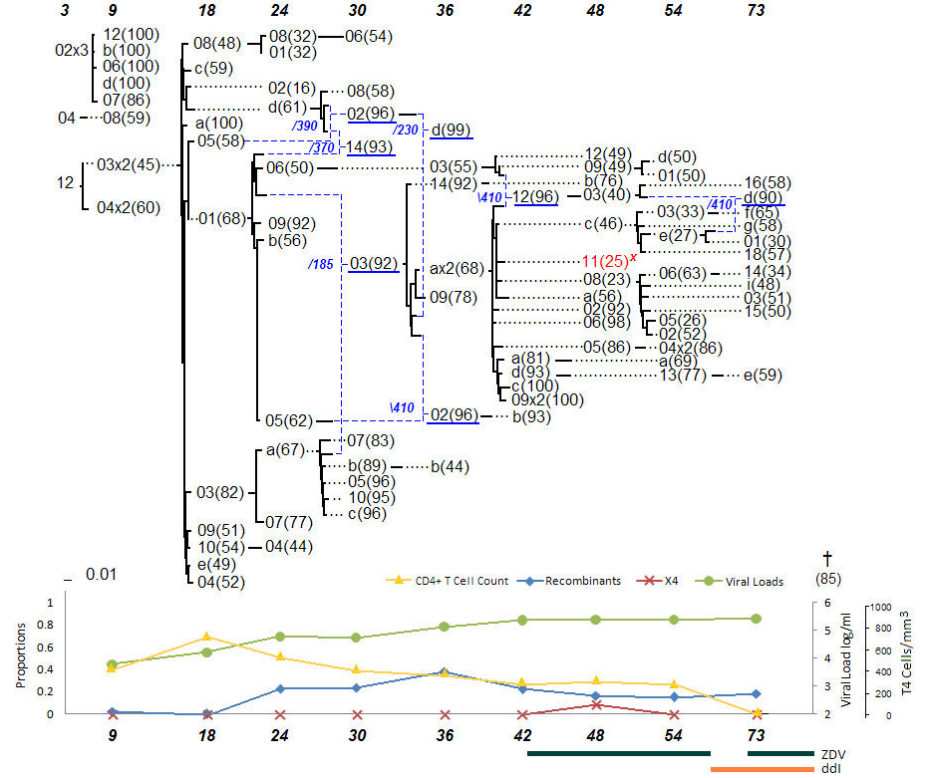
**Patient 6**.

**Figure 8:**
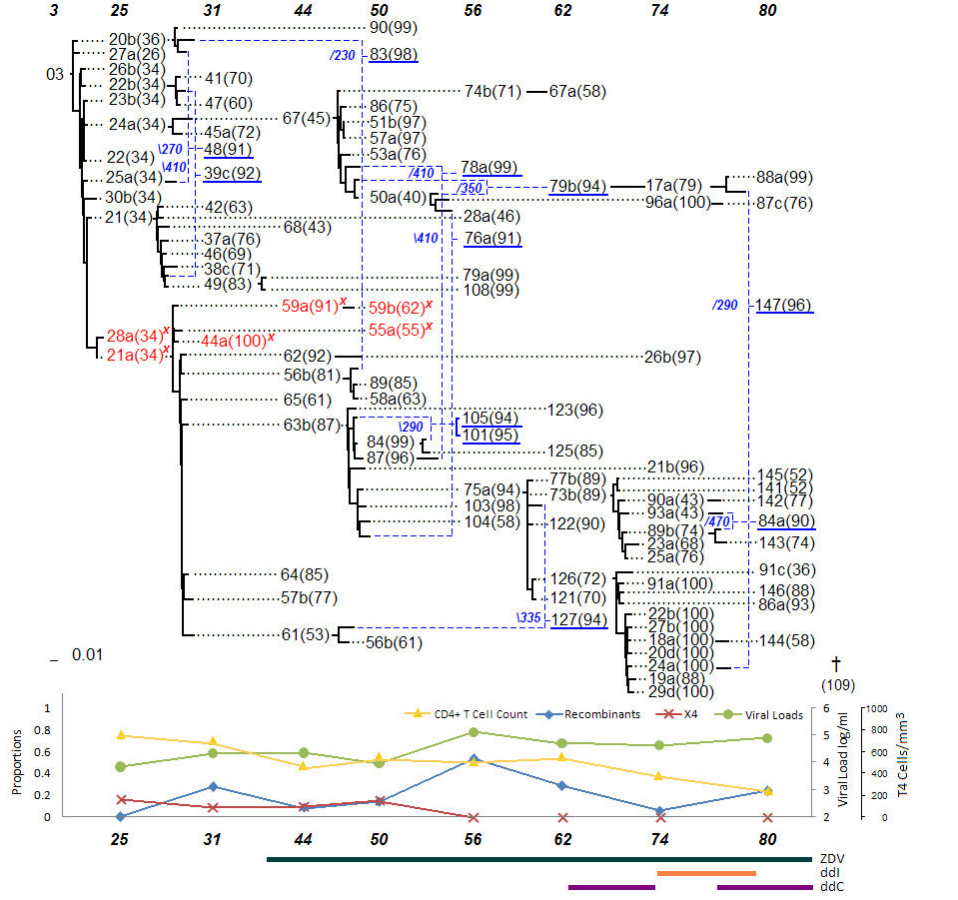
**Patient 7**.

Statistical analysis was performed by considering the data on all 8 patients (Figures 1, 2, 3, 4, 5, 6, 7 and 8). The networks of patients 1, 3, 6 and 7 showed only one single lineage with few recombination events. In contrast, it is remarkable that patient 5's network showed two separate lineages that coexisted until the 56 months sampling time point with no consensus recombination events linking them at any time point. Several sequences of patient 5 were predicted as recombinants by the different methods but no consensus was reached in these cases.

The networks revealed that the ancestors of the X4 strains were either recombinant or displayed strong recombination signals. A recombination signal is defined as an indication that different parts of a viral sequence may descend from different ancestors. In order to determine the significance of this pattern a score was devised to measure the recombination signal (RS) based on the average bootstrap support (BS) of the non-recombinant inferences, the number of recombinants (R) and the number of different ancestors (A) from the 6 results produced by analyzing two alignments (one gapped and one gap-balanced for subsets of the same data) by the 3 methods previously described. While only the consensus predictions are considered in the network construction, conflicting results may still indicate support for a recombination event, based on those three parameters. The strength of this support is measured by the recombination signal. The score is given by the following formula:



A low bootstrap support can indicate that either there is more than one possible ancestor, signaling a possible recombination event, or the determination of the ancestor is inconclusive. Results that differ in their ancestor prediction may also indicate a recombinant origin. A t-test confirmed that the ancestors of the X4 strains had a significantly higher recombination signal than the overall average recombination signal in all 8 patients (p = 0.003).

**Figure 9:**
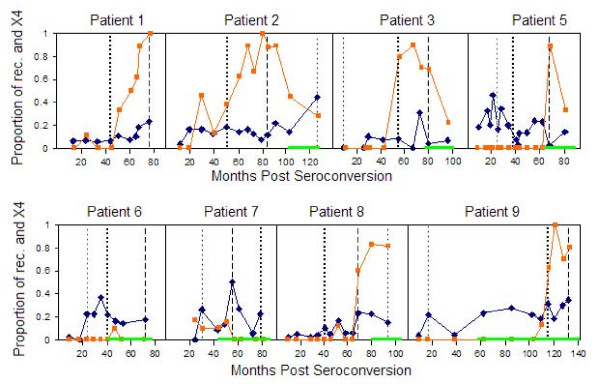
**Summary of recombination, X4 and viral load patterns**. The X-axis shows the sampling times in months post seroconversion, while the Y-axis shows the proportion of recombinants (blue diamond shapes) and X4 (orange square shapes) sequences. The light green thick line at the bottom along the X-axis shows the start and duration of antiviral drug therapy. The vertical dashed line indicates the highest peak in viral load levels; the dark dotted vertical line indicates the second highest peak, and the light dotted line the third highest peak in viral load levels. Patient 1 had only two viral load peaks, with a peak defined as a high point before a decline or at the last study time point.

In a further effort to investigate the relationship between clinical factors and evolutionary parameters we analyzed possible associations between the proportion of recombinants, the proportion of X4 mutants, the peaks in the viral load levels and the administration of antiretroviral drug therapy as shown in Figure 9. The information about the viral load levels were obtained from the authors of the previous study [21]. Several consistent patterns in the disease development of moderate HIV progressors were observed in a previous study: significant correlations were found between the years it took for the X4 virus to emerge and the time (since seroconversion) to peak virus diversity, between the time to peak prevalence of X4 and the time to divergence stabilization, and between the time to divergence stabilization and the time to T-Cell homeostasis failure [21]. In the current study we sought to find trends between the peak of viral load levels and the proportion of sequences identified as recombinants or as X4 mutants.

**Figure 10:**
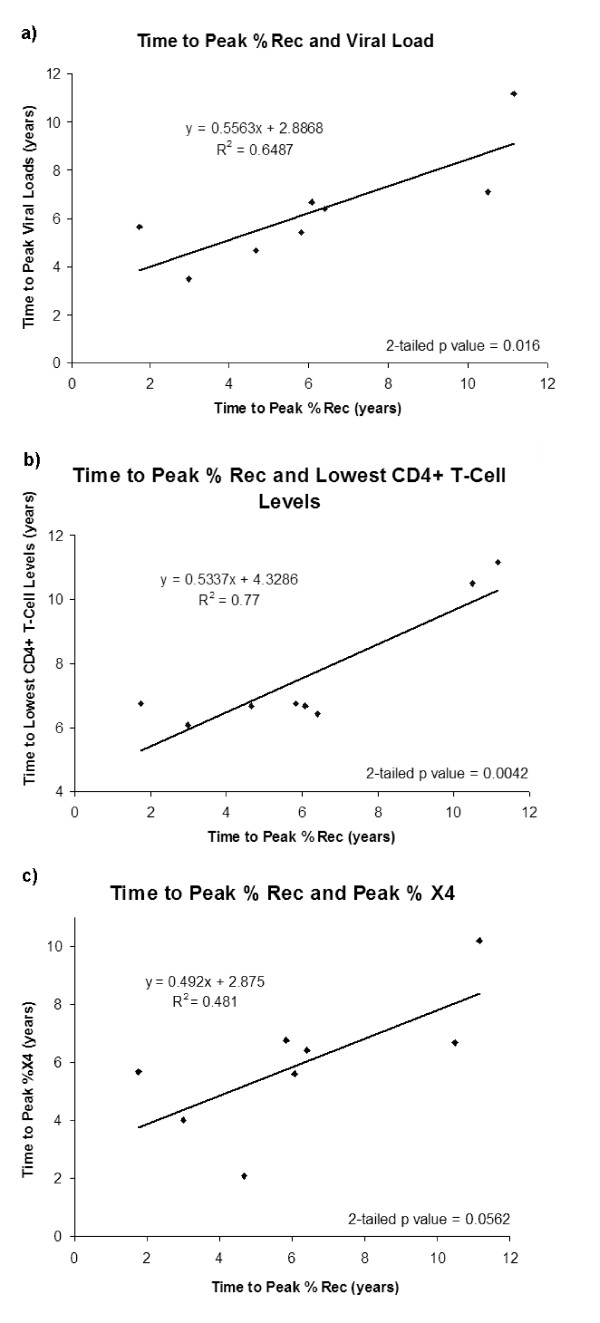
**Correlation Graphs**. Plots show relationships between (a) time to observed peak of viral load and the time to peak proportion of detected recombinants, (b) time to the lowest CD4+ T cells levels and the time to peak proportion of detected recombinants, (c) time to peak representation of X4 viruses and time to peak proportion of detected recombinants.

In this study, a consistent relationship was noted among the 8 patients between time variables involving clinical markers and proportion of recombinants as shown in Figure 10. The time from seroconversion to the observed peak of viral load and the time to peak proportion of detected recombinants were found to be significantly correlated (correlation coefficient r = 0.8, 2-tailed p = 0.016; if patient 5 was removed, the values were r = 0.89, p = 0.0072). An even higher correlation was found between the time to the lowest CD4+ T Cells levels and the time to peak proportion of detected recombinants (r = 0.87, 2-tailed p = 0.0042). The time to peak representation of X4 viruses and time to peak proportion of detected recombinants were also found to be correlated with a borderline significance (r = 0.69, 2-tailed p = 0.0562). In all cases, removing the outlier patient 5 improved the correlation coefficient and significance of the tests. Previous work showed that relative to failure of T-cell homeostasis, peak viral diversity occurred a mean of 2.2 years earlier with correlation analysis indicating that these two events where highly related [21]. No significant correlation was found between start of therapy and proportion of detected recombinants. The patterns involving putative reservoir virus could not be investigated statistically due to the low number of cases and the low bootstrap support of the non-recombinant evolutionary relationships.

We next asked if the star-like evolutionary pattern observed in the network of the five patients who died at the end of the study was associated with a faster disease progression. A star-like pattern is seen when many descendants share the same ancestor. A slight negative correlation was found between CD4+ T cell counts and the proportion of ancestors for each time period among 6 of the eight patients (with a mean slope of -0.19). A one sample t test comparing the mean slopes to a slope of zero returned a p-value of 0.0566.

Analysis of the sequences predicted as recombinants revealed that the average breakpoint position of X4 recombinant sequences was further to the right towards the V4 region of the *env *gene. The average breakpoint position for the non-X4 recombinant sequences was 373, while for the recombinant X4 sequences it was 427. A 1-tailed paired t-test returned a p-value of 0.0475. The analysis included predictions from the 6 result files per patient, far more than the few consensus recombinant sequences shown in the evolutionary networks. For example, for patient 5, there are many recombinant predictions but little consensus in most of them. No significant difference was found between breakpoint positions before and after drug therapy. The scatter plots of the breakpoint positions in relationship to the sampling time showed considerable diversity from patient to patient. The plots indicated a preference for the 300 to 500 bp region in all patients, but no particular *in vivo *hotspot for recombination could be observed. The distribution of recombination sites for all predicted recombinant sequences did not show discernible patterns across the C2 to C5 region with the exception of patient 1, for whom the breakpoint sites moved further to the left as the sampling time increased (r = 0.79, 2-tailed p < 0.001).

## Discussion

The Sliding MinPD method was used to analyze and represent the evolutionary relationships of HIV-1 sequence data sampled serially over a period of many years from the HIV-1 *env *region of eight patients. The network representation facilitated the locating of viral strains due to the temporal organization within the network and allowed for a better understanding of the evolutionary relationships of within-patient HIV strains. The evolution of HIV within-host environments is very different from between-host HIV evolution. There is strong evidence that natural selection is the driving force of within-host evolution. Within-host HIV phylogenies have a strong temporal structure, reflecting the successive fixation of advantageous mutations and the extinction of unfavorable lineages [2]. This can be observed in all the serial evolutionary networks presented here. Each of the networks obtained from the sequence data for the different patients appears to be qualitatively different and reveals different aspects of the evolutionary relationships among the data. Nevertheless, some surprising patterns could be observed.

### Patterns

A few of the recurring patterns observed in the evolutionary networks of the eight patients are summarized below and will be discussed in more detail in the next section:

▪ The network of the five patients who died at the end of the study displayed a starlike evolutionary pattern (approximate time of death in months appears in the network next to the symbol †).

▪ The founder strains of the lineage containing the X4 strains displayed significantly stronger recombination signals.

▪ Recombination events with early viral quasispecies suggest the resurgence of reservoir virus.

▪ Recombination events linking different major lineages were observed in the three surviving patients.

### Star-like evolution in the path to AIDS death

It is important to mention the role that the visualization of the results plays in understanding the different patterns of within-host viral evolution. The evolutionary networks made it easy to locate any viral strain due to the temporal positioning within the network, where strains sampled at the same sampling point were vertically aligned. The network representation can also encourage the pursuit of new research directions or generate new lines of inquiry. For example, knowing that five of the eight patients passed away after the analysis period, it was possible to ask whether any marked differences existed between the networks for the surviving and the deceased patients. As discussed in the results section, the networks of patients 2, 8 and 9 shared very similar characteristics such as the persistence of different lineages, unbalanced networks (as opposed to star-like networks where many branches extend in a balanced manner from a root node), recombination events between lineages and recombination events with much earlier viral strains. On the other hand, with the exception of patient 5, the networks of the other patients lacked these characteristics, with a distinctive star-like evolution of one single lineage from the onset of the study. Explosive growth generates characteristic 'star-like' phylogenies in which a single strain, the 'master sequence' in quasispecies theory, gives rise to a large population of diverse quasispecies with a spectrum of small mutational changes from the master sequence [28,29]. No literature could be found that linked star-like virus evolution with faster disease progression, but the association deserves to be studied more closely in the future.

### Evolution of the X4 mutants

The network representation allowed us to better understand the evolutionary roadmap of the X4 strains. Each of the patients had X4 strains, which are known to be associated with a higher rate of decline of CD4^+ ^T-cells, and therefore, a more rapid progression to AIDS [2]. Traditional phylogenetic tree models do not consider recombination events and in its presence may represent misleading evolutionary relationships. Evolutionary networks counteract that shortcoming by combining mutation and recombination models into one. The evolutionary networks presented here facilitate the investigation of patterns with regard to the emergence of the X4 strains. A recombinant origin for the X4 strains is suggested by all the patient's networks, and was found to be a statistically significant pattern as discussed in the results section. The founder strain of the lineage containing the X4 genotype was either a recombinant or had very low bootstrap support, which often happens when the methods are split on their choice of an ancestor. For example, sequence 34 m-81(38) in patient 1 had a low bootstrap support for its choice of ancestor (38%). Other sequences with low bootstrap support were 30 m-13(33) in patient 3, 56 m-k(39) in patient 5, 25 m-21a(34) in patient 7, 41 m-04(58) and 46 m-d(41) in patient 8. The sequences 51 m-19(93) in patient 2, 30 m-03(92) in patient 6, and 86 m-19(98) in patient 9 were predicted to be recombinant sequences. Crossover sites for predicted X4 recombinants were slightly to the left (toward the V4 *env *region) of crossover sites for other recombinant sequences.

The role of co-infection and recombination has received some attention in recent years and these findings may support the recombinant origin of the X4 strains [30-32]. It has been shown that frequent double infection can occur with CCR5- and/or CXCR4-tropic viruses, thereby generating opportunities for recombination to occur within viral populations [30].

### Remarkable Recombination Events

The serial evolutionary network representation allowed us to look for patterns present in the networks of the three surviving patients and absent in the other networks. As discussed earlier patients 8 and 9 have very similar network characteristics: separate lineages with linking recombination events and recombination events with strains from a much earlier time point. Patient 2 also displayed separate lineages and similar but less obvious patterns of recombination. In contrast to those three, Patient 5's network displays two long-lasting lineages with no linking consensus recombination events.

The presence of different types of HIV reservoir cells that harbor dormant virus has been discussed in many studies [26,27,33,34]. The persistence of reservoirs of HIV, including latently infected, resting CD4+ T-cells, which can give rise to infectious HIV after a period of latency, has posed a sobering challenge to the long-term control or eradication of HIV in infected individuals receiving highly active antiretroviral therapy (HAART). These reservoir viruses may co-infect a cell already infected with viruses from the current population, giving rise to new viral particles with 2 different parental RNA. It has been shown that HIV-1 coinfection and recombination foster rapid virus diversification and survival, and that productive cellular coinfection is not inhibited by cellular mechanisms [32]. In the current study, even though the framework allows for such inferences, many of the non-recombinant evolutionary relationships with putative escape reservoir virus had low bootstrap support, thus preventing us from finding support for such a claim.

In a recently proposed controversial model [35], it was suggested that clinical AIDS occurs only when immune-system cells were co-infected at a high rate – and the fast-killing virus strains won out. Our observations do not contradict this model, but we argue that co-infection may in some cases slow down disease progression when recombination is able to weed out the growing, but weak cytopathic effect of one of the infecting strains through the creation of more fit mosaic genomes. A fit virus in this context is a virus which survives by not killing its host. This could be a possible explanation for the observed patterns in the networks of patients 8 and 9. Recombination, like mutation, can have two outcomes: the new mosaic virus may propagate without destroying its host environment or it may propagate while leading to the extinction of its host. Maintaining low viral levels can ensure that mutation and recombination have less say in their evolutionary game of chance.

Figure 9, which summarizes patterns of recombination events as predicted by the Sliding MinPD method, suggests a linkage between the peaks in viral load levels and the peaks in the proportion of detected recombinants. The observation of an increase in the proportion of detected recombinant sequences may also, however, be attributed to other factors, such as an increase in viral sequence diversity. Sequence diversity was shown in a previous study to be highly related to the viral load levels [21]. Peaks in the viral load levels seem to correlate with the amount of recombinants at each sampling time. This is plausible in view of the mechanism of recombination in HIV-1, in which the likelihood of producing mosaic genomes increases with the number of diverse pairs of RNA strands that get packaged into a viral particle.

The current study did not estimate the Ka/Ks ratio of selection pressure, as it is known that recombination can affect the estimation of that measure. Current sitewise methods for detecting positive selection on gene sequences assume no recombination. When this assumption is violated it can lead to incorrect detection of sites undergoing positive selection [36]. A recent Bayesian MCMC method tries to solve the problem by estimating variation in selection pressure along a sequence in the presence of recombination [37] and will be used in future research.

## Conclusion

The serial evolutionary networks of the 8 patients revealed different aspects of the within-host evolution of the HIV virus. Among the networks for the 8 patients we found several consistent patterns that are of evolutionary significance with potential impact on the development of new anti-retroviral drugs. The visualization of the results played an important role in understanding the different patterns of within-host viral evolution. The network representation suggested new avenues of research and lead to statistical analysis that connected our observations with known facts and previously published results on the evolution of the HIV-1 virus. The serial evolutionary network proved to be an effective and useful tool, able to produce noteworthy results, as well as generate important and productive new lines of inquiry in the study of viral evolution.

The main line of inquiry was aimed at studying the origin of the disease associated X4 strains. As previously discussed, the putative founder strains of the X4 population are most likely a result of recombination events in large and diverse viral populations. Our results suggest that evidence of recombination increases with higher viral loads. On the other hand, recombination between strains from different lineages or between reservoir viruses and the viruses from the dominant population were more prevalent in the asymptomatic patients. A star-like topology could be observed in the networks of four of the five patients who died of AIDS. As mentioned in the earlier study [21], it is not clear whether the patterns and sequence of events observed with the current selection of patients can be generalized to patients with a slower or faster disease progression rate. Despite these concerns, this study offers new insights into the role of recombination on HIV evolution within a patient and a practical methodology for making inferences from these evolutionary networks.

Recent sequencing technologies such as that from 454 Life Sciences have made it possible to quickly and cheaply generate a large number of short clonal transcripts (~100–400 bp). The inevitability of large-scale HIV within-host sequences data in the near future makes it critical to develop analytical methods of the kind presented in this paper. We expect an abundance of new clonal sequences from longitudinal studies, which can be used in the reconstruction of serial evolutionary networks for a much larger number of patients and can contribute to the development of tools for HIV patient surveillance and prognosis. Sliding MinPD, which is the most efficient and accurate programs for this kind of data, should prove extremely useful in such analyses. Although modern HIV combination drug therapy has been successful at suppressing viral replication to <50 c/ml in patients who adhere to treatment, viral replication has been shown to persist in viral reservoirs and inconsistent adherence to treatment can lead to drug resistance. Moreover, in developing countries, infected individuals with limited access to drugs face an increased risk of developing AIDS. It therefore continues to be of foremost importance to understand the evolutionary process of HIV within a patient to aid in the design of effective drug treatments and in the evaluation of patient prognosis.

## Authors' contributions

PB designed the study, carried out the statistical analysis and drafted the manuscript. GN participated in the design of the study and writing of the manuscript. All authors read and approved the final manuscript.
